# Successful Multimodal Treatment of Pancreatic Cancer With Extensive Superior Mesenteric Vein Thrombosis Utilizing Chemotherapy Combined With Direct Oral Anticoagulant

**DOI:** 10.7759/cureus.53657

**Published:** 2024-02-05

**Authors:** Kei Harada, Takahisa Fujikawa, Taisuke Matsuoka, Yusuke Uemoto, Norio Emoto

**Affiliations:** 1 Surgery, Kokura Memorial Hospital, Kitakyushu, JPN; 2 Surgery, Ijinkai Takeda General Hospital, Kyoto, JPN

**Keywords:** borderline resectable pancreatic cancer, superior mesenteric vein thrombosis, conversion surgery, antithrombotic therapy, gemcitabine plus nab-paclitaxel therapy

## Abstract

It is well known that portal vein thrombosis (PVT) sometimes occurs in pancreatic cancer (PC). However, no effective treatment plan for PVT in PC patients has yet been proposed. We experienced a successfully treated case of borderline resectable pancreatic cancer (PC-BR) with extensive superior mesenteric vein thrombosis utilizing intensive chemotherapy combined with direct oral anticoagulant. The thrombus disappeared and the tumor shrank, enabling curative surgery, and long-term survival for more than five years has been achieved. We report this successful case that we experienced as an option for the treatment of PC-BR with PVT in the future era when multimodal treatment is important.

## Introduction

Pancreatic cancer (PC) is a typical intractable malignant tumor and is one of the most prothrombotic tumors, with thrombotic complications occurring at a rate of up to 30% [1]. Portal vein thrombosis (PVT) associated with PC is frequently discovered incidentally and may contribute to an increased risk of complications and a poor prognosis [2]. Due to insufficient evidence, no effective treatment plan for PVT in PC patients has yet been proposed [3].

The underlying diseases of PVT are known to include portal hypertension such as liver cirrhosis, malignant tumors, intra-abdominal infection and inflammation, and genetic diseases that cause coagulation abnormalities [3]. Concerning PVT related to abdominal malignancy such as PC, compression of the portal vein (PV) or superior mesenteric vein (SMV) and subsequent decreased blood flow caused by the invasive tumor are presumed to be the triggers for thrombosis formation [4]. Anticoagulation therapy has generally been advised in the presence of PVT in patients with malignancy, although the safety and feasibility of intensive chemotherapy combined with the use of direct oral anticoagulants (DOACs) in the treatment of cancer-related PVT remain unknown [5].

It is suggested that a treatment case utilizing intensive chemotherapy combined with DOAC and allowing for curative surgery will be useful in this modern era, where multimodal treatment is becoming more prevalent. Herein, we report on a successfully treated case of borderline resectable pancreatic cancer (PC-BR) with extensive superior mesenteric vein thrombosis utilizing intensive chemotherapy combined with DOAC.

## Case presentation

**Figure 1:**
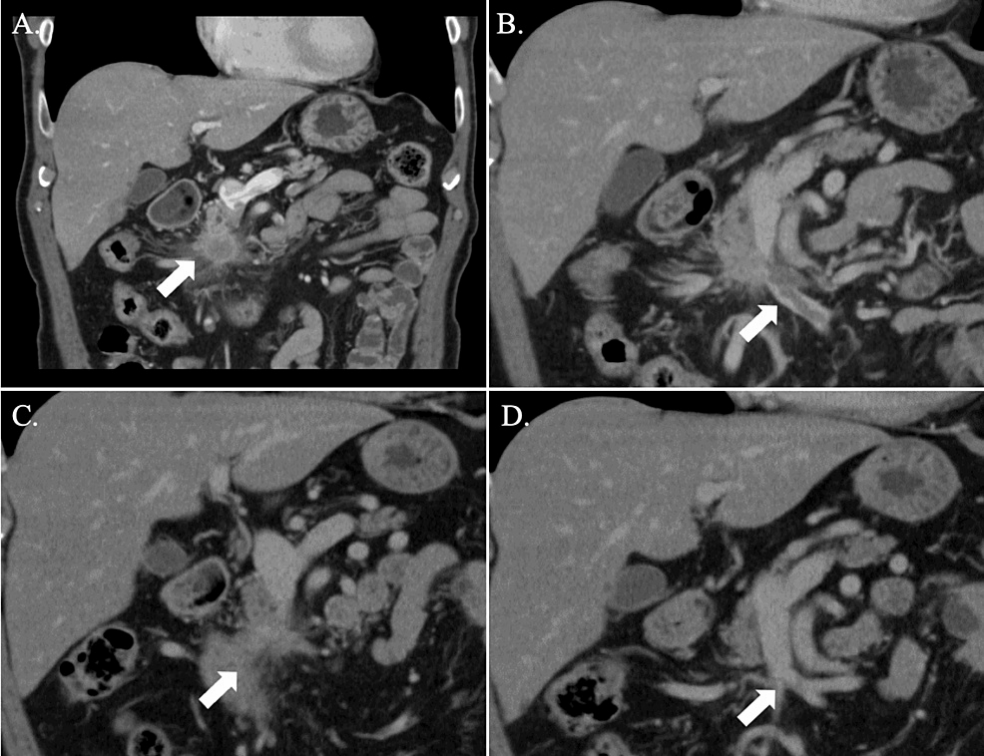
Contrast-enhanced CT images of pancreatic head cancer in the current case before and after the intensive chemotherapy combined with a DOAC. (A) Before the therapy, a nodule with a diameter of around 22 mm and low contrast enhancement was present in the pancreatic head (white arrow). (B) An extensive thrombus was also found in the SMV and J1V (white arrow). (C) After the therapy, main tumor shrank and only soft shadow remained (white arrow). (D) Thrombosis within SMV and J1V had completely disappeared (white arrow). CT; computed tomography, DOAC; direct oral anticoagulant, SMV; superior mesenteric vein, J1V; vein of the first jejunum.

A 70-year-old man came to our hospital with anorexia and upper abdominal discomfort. Previous medical history revealed nothing unusual, but he lost about 9 kg of body weight in six months. In addition, no obvious abnormal findings were found in upper and lower gastrointestinal endoscopies. No obvious abnormal findings were observed in blood biochemical tests. An enhanced computed tomography (CT) scan showed a 22-mm low-density nodule with distal main pancreatic duct dilatation in the pancreatic head (Figure 1A). Although the nodule was distant from the celiac artery (CA) and superior mesenteric artery (SMA), extensive involvement of the SMV was observed. There was also an extensive defect in the SMV and vein of the first jejunum (J1V) distal to the tumor, suggesting severe SMV thrombosis (Figure 1B). The defect was relatively poorly contrasted and bead-shaped, and was extending widely within the SMV, suggesting severe SMV thrombosis, rather than tumor thrombus. There were no findings that suggested distant metastasis. Initial tumor marker levels were as follows: carcinoembryonic antigen (CEA) was 1.7 ng/mL, carbohydrate antigen (CA19-9) was 23 U/mL, duke pancreatic mono-clonal antigen type 2 (DUPAN-Ⅱ) was 25 U/mL, and s-pancreas-1 antigen (SPAN1) was 15.2 U/mL.

A diagnosis of adenocarcinoma was obtained by endoscopic ultrasound-fine needle aspiration (EUS-FNA). The patient was diagnosed with PC-BR with extensive SMV thrombosis, and treatment with gemcitabine (GEM) + nab-paclitaxel (nab-PTX) combined with a DOAC (edoxaban, 30 mg/day) as antithrombotic therapy was initiated and continued for four months. GEM and nab-PTX were given at a dose of 1730 mg (1000 mg/m2) and 215 mg (125 mg/m2), respectively. As a result, the main tumor shrank, and only a soft shadow remained (Figure 1C). Furthermore, the SMV thrombus disappeared (Figure 1D). Response Evaluation Criteria in Solid Tumors (RECIST) determined the partial response.

**Figure 2:**
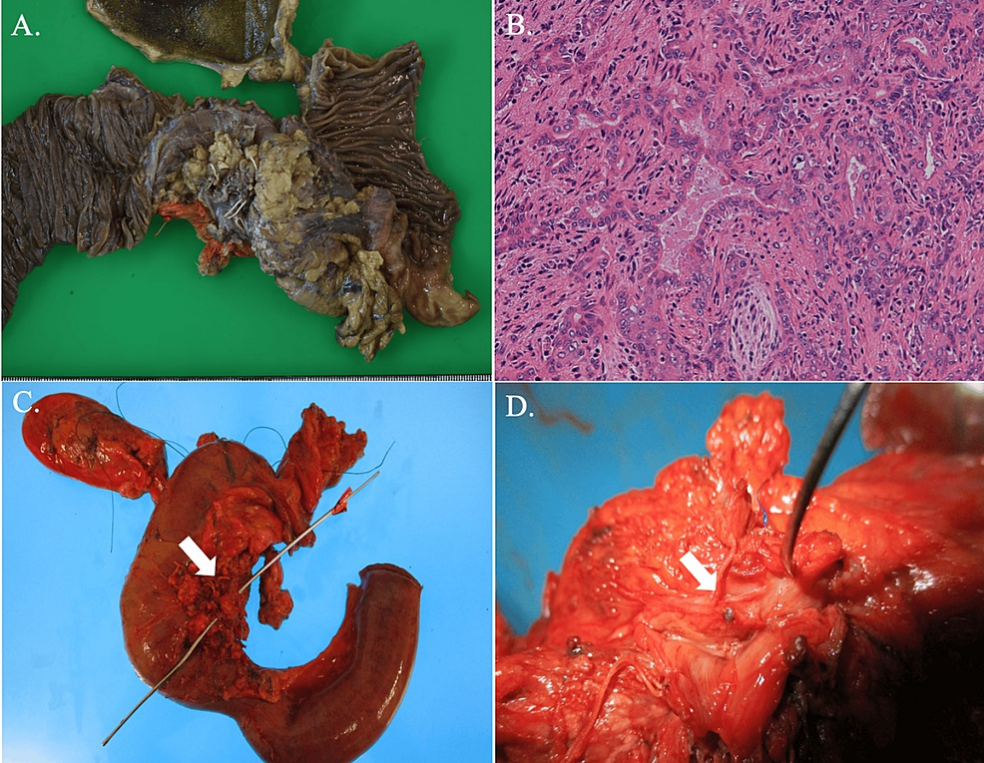
The resected specimen and histopathological findings of the tumor in the current case. (A) A 30 × 20 mm mass with unclear borders was found in the pancreatic head within the resected specimen. (B) Histopathological examination revealed moderately differentiated tubular adenocarcinoma. A cancer invasion into the PV and retropancreatic nerve plexus was observed. (C) PV and SMV were involved in the tumor (white arrow indicates the area where the tumor has invaded the PV). (D) An enlarged view of the area where the tumor invaded the PV (the PV was cut and the inside of the PV was shown). The tumor invasion was observed more clearly (white arrow). PV; portal vein, SMV; superior mesenteric vein.

At that point, curative resection was considered possible, and surgery was planned. During the operation, we found that the pancreatic head tumor invaded SMV, but no other unresectable factor was observed, and subtotal stomach-preserving pancreatoduodenectomy (SSPPD) combined with SMV resection under general anesthesia was successfully performed (Figure 2A-2D). SMV was reconstructed by end-to-end anastomosis without using a vein graft. During postoperative hospitalization, the patient had a mildly low nutritional intake, which gradually improved over time, and was discharged home on postoperative day 28 without any obvious complications. Pathological findings were adenocarcinoma, moderately differentiated type (mod), pT3N0M0 (Figure 2B). Cancer infiltration was observed in the portal vein and retropancreatic nerve plexus. The therapeutic effect was Grade 1b. As postoperative adjuvant chemotherapy, tegafur, gimestat and otastat potassium combined drug (S-1, 100 mg/day) was administered for six months, although edoxaban has been discontinued since the surgery. The patient has been alive and doing well for more than five years with no recurrence after surgery.

In reporting the case, we respected ethical considerations, including the protection of personal privacy, and provided sufficient informed consent to the patient.

## Discussion

PC is known to have the poorest prognosis among gastrointestinal cancers [6]. There are also reports that the five-year survival rate is less than 10% [7]. On the other hand, as in our case, there are increasing reports of patients with PC-BR who were able to undergo curative surgery through multidisciplinary treatment and achieve long-term survival [8,9]. Regarding the relationship between malignant tumors and thrombosis, PC has been linked to an increased risk of venous thrombosis (VT), and it is well known that PVT occurs more frequently than in other malignancies [10]. Insufficient blood flow is recognized as one of the causes of PVT, and because PC is anatomically close to PV, it is likely to be susceptible to infiltration and displacement, leading to poor blood flow [11]. However, no specific treatment regimen with effective evidence is currently available for PVT with PC [11,12].

Due to strong evidence of increased efficacy and safety, antithrombotic therapy with low molecular weight heparin or unfractionated heparin has generally been advised in the presence of PVT or SMV thrombosis in patients with malignancy [12,13]. Recently, there have been more reports that DOACs are not worse than heparin for VT, even though the evidence is still limited [13]. DOACs also seem to be a good option for antithrombotic therapy in cancer patients [14]. All types of DOACs are easy to use because they are simple to administer, don't require monitoring or dose adjustments, and have few drug-drug interactions [14]. In the current case, we used edoxaban for antithrombotic therapy combined with intensive chemotherapy for four months, and subsequently, the SMV thrombosis completely disappeared. Chemotherapy was also successful, making curative resection possible.

**Figure 3:**
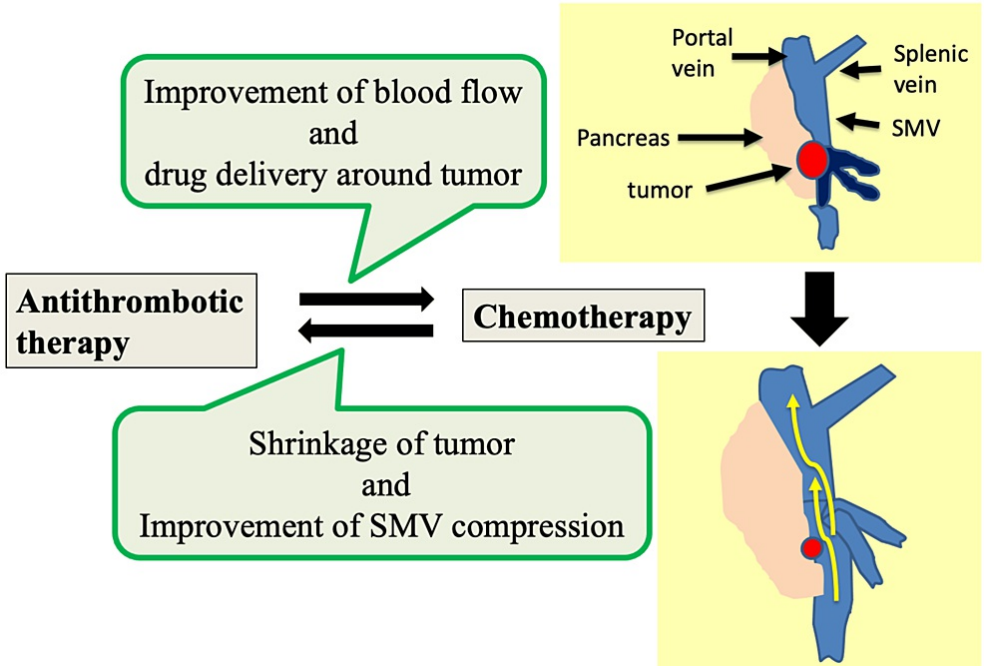
The speculated mechanism of combination therapy using intensive chemotherapy and anticoagulation therapy for PC-related PV and/or SMV thrombosis. PC; pancreatic cancer, PV; portal vein, SMV; superior mesenteric vein This figure is the authors' own creation.

Concerning the combination therapy using both intensive chemotherapy and anticoagulation therapy for locally advanced pancreatic cancer, the mechanism is speculated as follows (Figure 3). First of all, thrombosis typically begins in large veins at the site of compression and progresses peripherally to involve smaller vessels in intra-abdominal diseases such as PC [15]. This mechanism of thrombus development helps explain the interaction between antithrombotic therapy and chemotherapy in this case. Antithrombotic therapy improves blood flow and drug delivery. The effect of chemotherapy was enhanced, the tumor shrank, and the pressure on the SMV was resolved. Blood flow around the tumor is also improved, and the common mechanism of thrombus formation associated with PC is resolved, like in the current case.

As in the current case, we believe it is important to consider appropriate treatment for each patient in this modern era, where multimodal treatment is becoming more prevalent.

## Conclusions

We successfully treated PC-BR with extensive SMV thrombosis utilizing intensive chemotherapy combined with DOAC. As a result, the thrombosis completely disappeared and the tumor shrank, enabling curative surgery. Furthermore, long-term survival for more than five years has been achieved. It was suggested that the combination of intensive chemotherapy and DOAC therapy is one of the preferred options for the treatment of PC-BR with extensive PVT.
